# Beyond Iron Deficiency: An Atypical Peripheral Smear Revealing Hemoglobin C in an Adolescent

**DOI:** 10.7759/cureus.104722

**Published:** 2026-03-05

**Authors:** Mahmoud Hamidine, Mehdi Awati, Aziki Mehdi

**Affiliations:** 1 Department of Hematology and Oncology, Avicenna Hospital, Medical School of Cadi Ayyad University, Marrakech, MAR; 2 Department of Biological Hematology, Avicenna Hospital, Medical School of Cadi Ayyad University, Marrakech, MAR; 3 Department of Biological Hematology, Avicenna Hospital, Marrakech, MAR

**Keywords:** anemia, hemoglobin c, hemoglobinopathy, iron deficiency, peripheral blood smear

## Abstract

Hemoglobinopathies are among the most common inherited disorders worldwide and represent a significant public health concern. The clinical presentation varies depending on genetic status--heterozygous or homozygous--and may involve structural abnormalities, defects in hemoglobin synthesis, or both. Despite their prevalence, hemoglobinopathies remain underdiagnosed, particularly when masked by more common causes of anemia. We report a 14-year-old male adolescent with no significant past medical history, referred for evaluation of microcytic hypochromic anemia. The clinical examination revealed pallor and mild splenomegaly, while laboratory tests showed low serum ferritin, decreased folate levels, and mild hyperbilirubinemia. Peripheral blood smear demonstrated target cells, anulocytes, dacryocytes, and occasional crystal-like erythrocytes. Hemoglobin electrophoresis and high-performance liquid chromatography (HPLC) confirmed a heterozygous hemoglobin C (HbC), previously unrecognized in the patient and his family. This case highlights the importance of considering hemoglobinopathies in the differential diagnosis of microcytic hypochromic anemia, even when iron deficiency appears to be the most likely cause. It emphasizes the diagnostic value of peripheral blood smear, an investigation often overlooked in routine practice, which was crucial for identifying heterozygous HbC in this patient. Beyond the individual case, it underscores the need for systematic diagnostic approaches, genetic counseling, and family screening to reduce underdiagnosis and mitigate the public health impact of hemoglobinopathies.

## Introduction

Hemoglobinopathies are the most prevalent monogenic disorders worldwide [1] and represent an increasing public health challenge [2]. Originally confined to sub-Saharan Africa, the Mediterranean, the Middle East, and parts of Asia [3], their distribution has become global as a result of population migration [4,5]. Currently, an estimated 7% of the world’s population carries a pathogenic hemoglobin variant, leading to 300,000-400,000 affected newborns each year [6,7].

These, caused by amino acid substitutions in globin chains, most commonly affecting the β-globin chain, or from synthetic (quantitative) defects, resulting in hemoglobinopathies, arise either from structural (qualitative) defects or thalassemia syndromes due to partial or complete absence of one or more globin chains (α, β, δ, or γ) [8,9]. Some individuals present with compound hemoglobinopathies [10], combining structural and/or quantitative abnormalities, which may lead to variable clinical severity [11,12].

Although many hemoglobin variants are clinically silent, their detection can be masked by coexisting conditions, such as deficiency-related disorders, which may dominate the clinical presentation and delay recognition of the underlying hemoglobinopathy [3,13].

Here, we report the case of a 14-year-old adolescent initially suspected to have iron deficiency anemia, in whom a detailed hematologic assessment revealed heterozygous hemoglobin C (Hb C).

## Case presentation

A 14-year-old adolescent, the first of two siblings, with no significant medical history, was referred for the evaluation of microcytic hypochromic anemia, initially presenting with fatigue and exertional dyspnea during school sports. On admission, examination revealed pallor, mild jaundice, and a palpable spleen tip, with no lymphadenopathy or bleeding manifestations.

The Initial laboratory tests showed hemoglobin 11.8 g/dL, mean corpuscular volume (MCV) 71 fL, mean corpuscular hemoglobin concentration (MCHC) 27 g/dL, serum ferritin 3.2 ng/mL, and folate 3.1 ng/mL (Table 1).

**Table 1:** Laboratory results during follow-up LDH: Lactate Dehydrogenase; HAV: Hepatitis A Virus; HBV: Hepatitis B Virus; HCV: Hepatitis C Virus; NR: Not Realized

Parameters	Initial values	Values ​​after 4 months of deficiency correction	References values units
Hemoglobin	11.8	13.0	12 – 16 g/dL
Mean Corpuscular Volume	71	80	80 – 100 fL
Mean Corpuscular Hemoglobin Concentration	27	33	32 – 36 g/dL
Serum Ferritin	3.2	45	15 – 150 ng/mL
Serum Folate	3.1	NR	> 4.0 ng/mL
Liver Function (Bilirubin)	Mild Hyperbilirubinemia	NR	< 1.2 mg/dL
Thyroid Function	Normal	NR	0.27 – 4.20 µUI/ml
LDH	NR	NR	135 – 225 U/L
Haptoglobin	NR	NR	0.4 – 2.2 g/L
Viral Serologies (HBV, HAV, HCV)	Négative	NR	Négative

**Figure 1:**
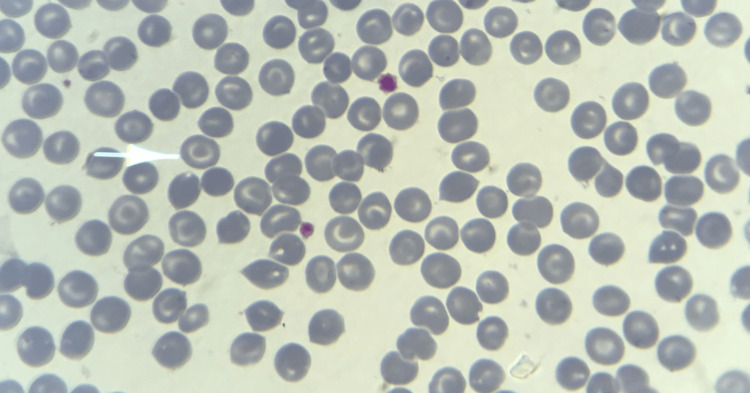
Peripheral blood smear at 1000x magnification and after Giemsa staining, showing target cells

**Figure 2:**
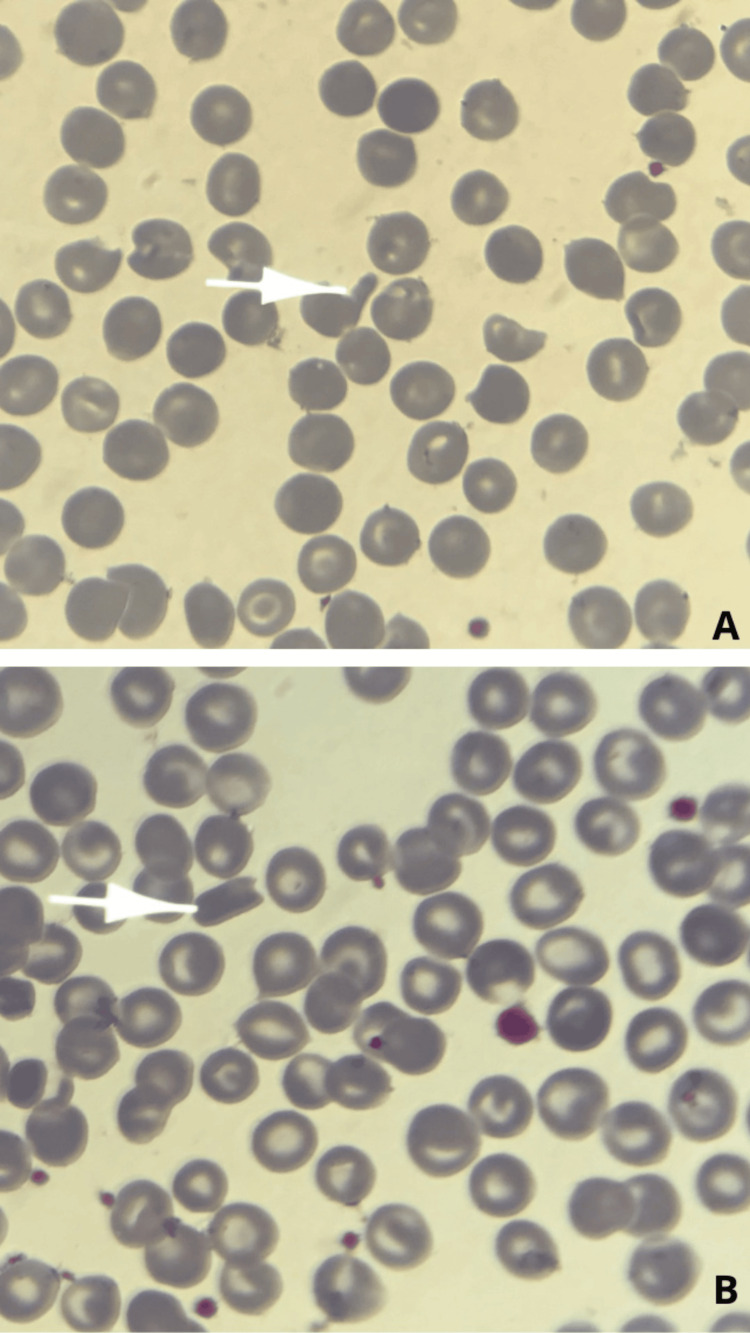
A and B representing a peripheral blood smear at 1000x magnification and Giemsa staining, showing rod cells

A repeat evaluation confirmed persistent microcytic hypochromic anemia (Hb 10.9 g/dL). Peripheral blood smear demonstrated anisopoikilocytosis, including target cells, dacryocytes, anulocytes, and occasional rod- or crystal-shaped erythrocytes (Figures 1, 2, panels A and B). Liver function tests revealed mild hyperbilirubinemia, whereas thyroid function and viral serologies (HBV, HAV, HCV) were normal (Table 1).

**Figure 3:**
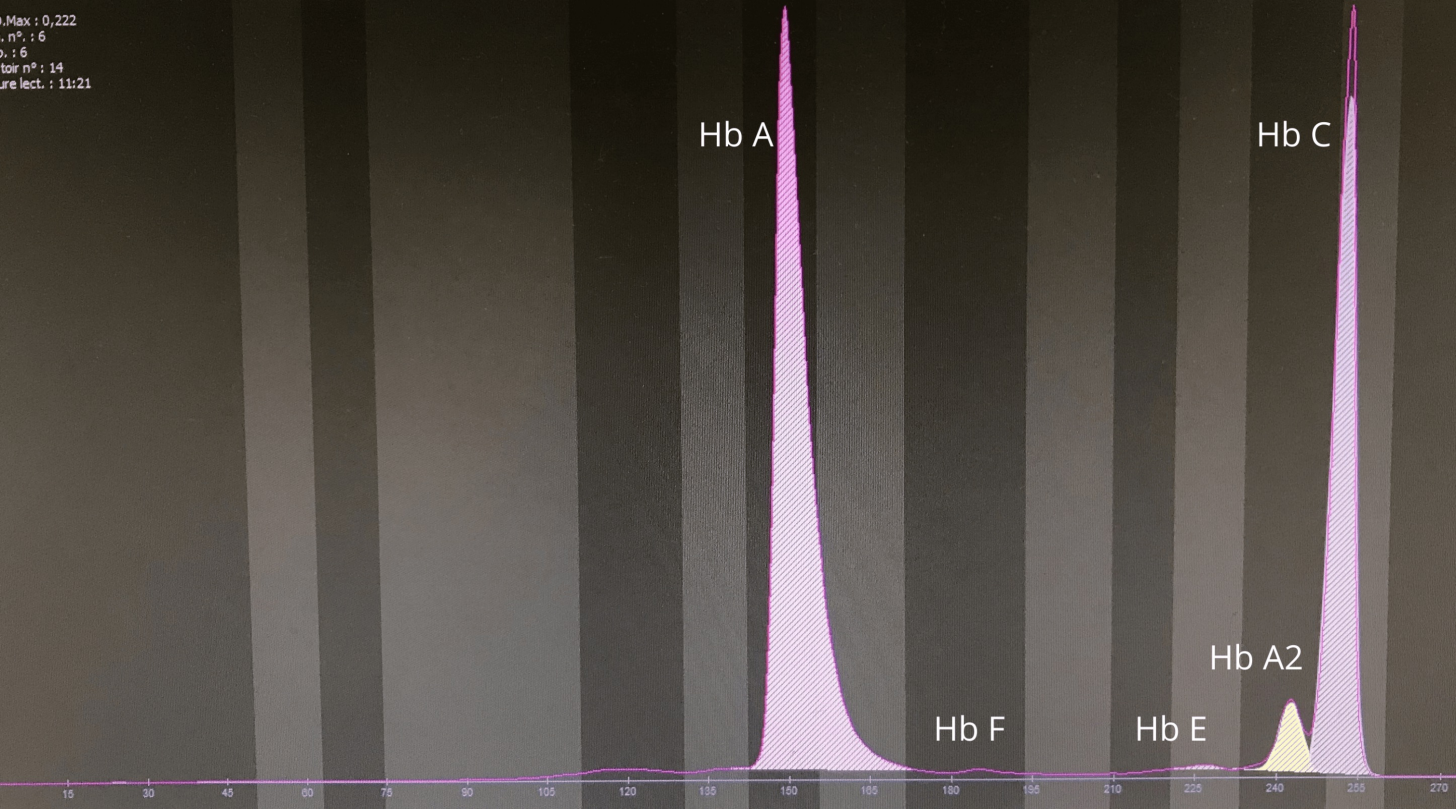
Electrophoretic profile of the patient by the capillary technique, showing hemoglobinosis C in the heterozygous state Hb A: 60.5% (Ref. range 95-98); Hb F: 0.3 (Ref. range <1); Hb E: 0.3 (Ref. range 0); Hb A2: 4.1 (Ref. range 2-3); Hb C: 35.1 (Ref. range 0)

Based on peripheral smear findings, capillary hemoglobin electrophoresis revealed elevated HbA₂ and heterozygous HbC (Figure 3). A low HbE fraction was also detected, raising initial suspicion for a compound heterozygous hemoglobinopathy (Hb C/Hb E).

**Figure 4:**
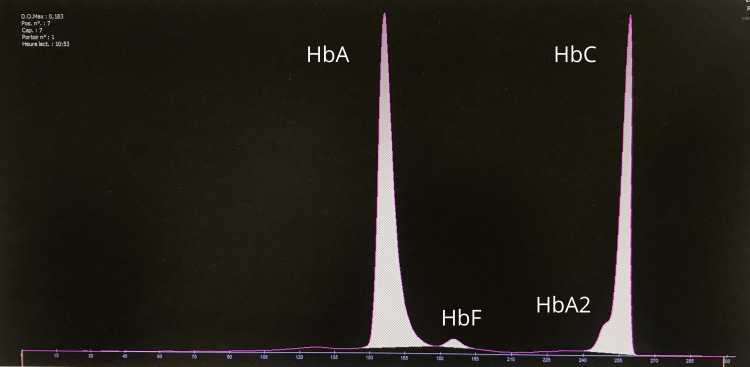
Electrophoretic profile of the mother by the capillary technique, showing hemoglobinosis C in heterozygous state Hb A: 60.1 (Ref. range 95-98); Hb F: 1.5 (Ref. range <1); Hb A2: 2.9 (Ref. range 2-3); Hb C: 38.4 (Ref. range 0)

The patient was initiated on oral iron and folate supplementation. A family screening revealed heterozygous Hb C in the mother (Figure4), while the father and sibling were unaffected.

**Figure 5:**
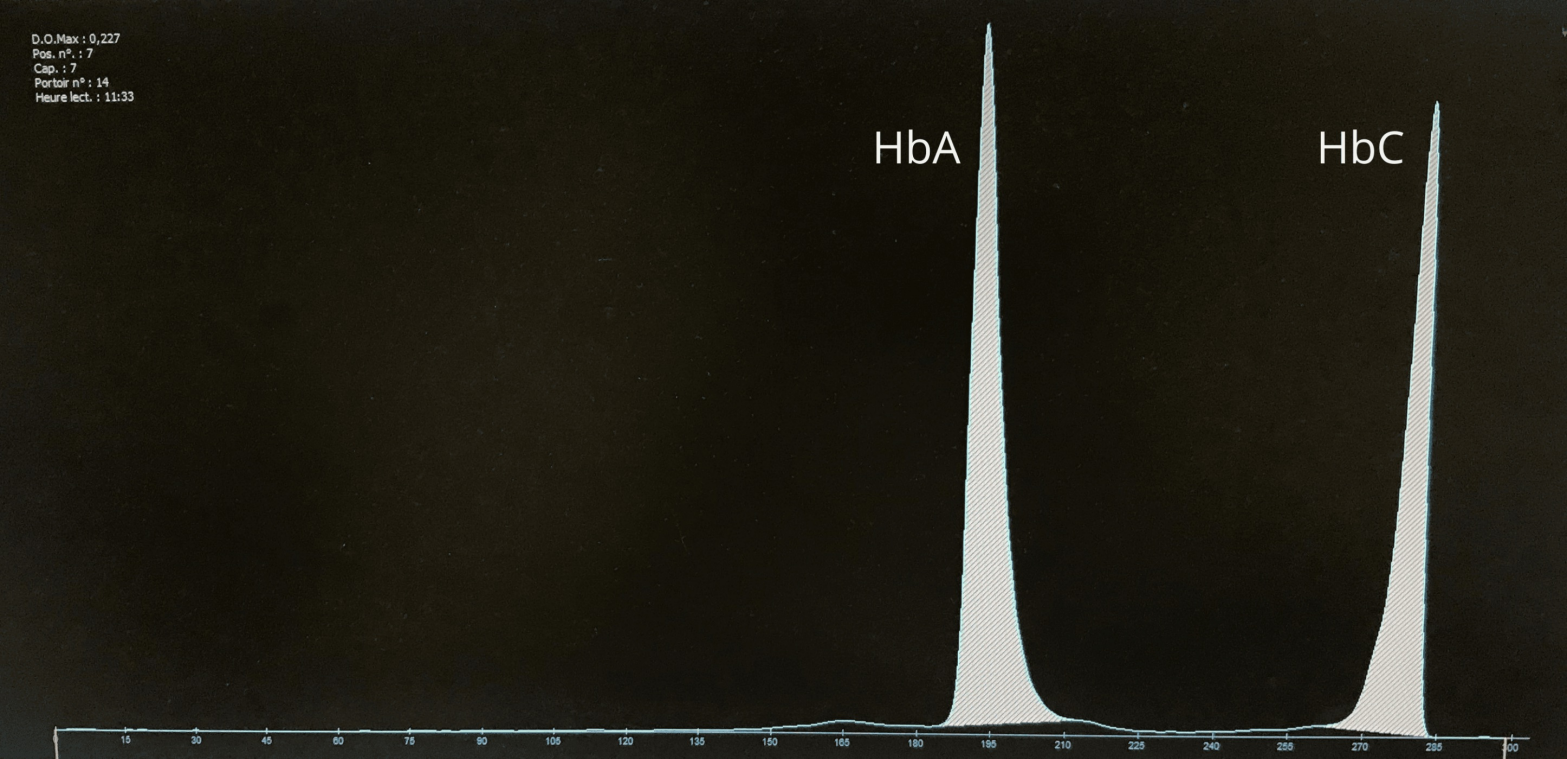
Electrophoresis of the patient's hemoglobin by capillary technique after iron and folic acid supplementation Electrophoresis of the patient's hemoglobin after iron and folate supplementation, showing the disappearance of the hemoglobin E trait, excluding a compound heterozygous state. Hb A: 59.3 (Ref. range 95-98); Hb C: 40.47 (Ref. range 0)

The clinical improvement was accompanied by normalization of hematological parameters: hemoglobin 13 g/dL, MCV 80 fL, MCHC 33 g/dL, and serum ferritin level 45 ng/mL(Table 1). A repeat hemoglobin analysis using electrophoresis and high-performance liquid chromatography (HPLC) confirmed normalization of HbA₂ and persistence of heterozygous Hb C, with disappearance of the Hb E fraction, excluding a compound heterozygous state (Figure 5).

## Discussion

HbC was first described in 1951 by Itano and Neel, who identified its distinctive electrophoretic migration pattern in symptomatic patients [11]. It results from a single-point mutation in the β-globin gene, substituting glutamic acid with lysine at position 6 (β6 Glu→Lys). This structural alteration promotes potassium efflux and red cell dehydration, leading to mildly microcytic, often normochromic erythrocytes. At higher intracellular concentrations, HbC tends to crystallize into rhomboid inclusions, which impair membrane ion transport, increase blood viscosity, and decrease erythrocyte deformability. These changes are associated with a predominantly prothrombotic rather than hemolytic profile [3,12].

Epidemiologically, the prevalence of HbC is estimated to be less than 1% in Central Africa and is rarely reported in East Africa [3,12]. It has also been identified in Southern Europe, particularly in Italy and Turkey [6]. Outside these regions, HbC remains relatively frequent among individuals of African descent, with a prevalence of approximately 2.4 per 100,000 in African Americans in the United States [13] and around 3.5% in Caribbean populations. Sporadic cases have additionally been documented in the Arabian Peninsula, the Balkans, and Sicily.

Clinically, heterozygous carriers (HbAC) are usually asymptomatic, though mild microcytosis with occasional target cells may be observed. In contrast, homozygous HbC disease is typically associated with chronic moderate hemolysis, sometimes complicated by hypersplenism, cholelithiasis, folate deficiency, musculoskeletal pain, retinopathy, and, less commonly, dental abnormalities [14,15]. Rare vaso-occlusive episodes have been reported, particularly during pregnancy [14]. Compound heterozygous states generally result in more severe phenotypes, such as HbSC disease, which closely resembles homozygous sickle cell disease, or HbC/β-thalassemia, which manifests as an intermediate thalassemic syndrome [3,12].

The finding of HbC in our 14-year-old patient highlights the incidental detection of hemoglobinopathies during the investigation of apparently benign hematological abnormalities. The initial clinical picture, with hypochromic microcytic anemia and low ferritin level, strongly suggested iron deficiency anemia. However, the identification of morphological abnormalities on the peripheral blood smear (target cells and occasional red blood cell crystals) prompted further investigations by hemoglobin electrophoresis and high-performance liquid chromatography (HPLC). These analyses confirmed heterozygous HbC and a transiently low HbE fraction, which normalized after iron and folate supplementation.

This observation is consistent with several international studies, in which hemoglobinopathies such as HbC, hemoglobin SC (HbSC), or Hb Lepore have been discovered incidentally during hematological evaluations for anemia, fatigue, or musculoskeletalpain [16,17], or during routine laboratory analyses, including HbA1c measurements in asymptomatic individuals [18]. Such cases highlight the importance of considering hemoglobinopathies in the differential diagnosis of microcytic anemia and underline the diagnostic value of the blood smear, especially when laboratory abnormalities appear discordant with a simple nutritional deficiency.

## Conclusions

Microcytic anemia is not always synonymous with iron deficiency alone, even if clinical and laboratory data are supportive, and the likelihood of an underlying hemoglobinopathy is always present. In cases of coexistence, diagnostic errors can occur, suggesting a composite hemoglobinopathy. Correction of deficiencies, rigorous interpretation of electrophoretic results, and confirmation with other analytical techniques are essential to avoid these errors.

This case highlights the integration of epidemiological, clinical, and laboratory data in the assessment of hemoglobinopathies and underscores the importance of early detection, not only for appropriate patient management but also to guide family screening and genetic counseling. Such an approach is essential to prevent the development of serious composite forms and anticipate potential clinical complications.
